# Prevalence of mineralization in the para-aural region in dogs with and without ear disease

**DOI:** 10.3389/fvets.2026.1755837

**Published:** 2026-04-02

**Authors:** Man-Hei Ma, Janet Bain, Tim Sparks, Abby Caine

**Affiliations:** 1 Dick White Referrals, Cambridgeshire, United Kingdom; 2 Waltham Petcare Science Institute, Leicestershire, United Kingdom; 3 Department of Clinical Veterinary Medicine, Cambridge, United Kingdom

**Keywords:** canine, computed tomography, middle ear disease, mineralization, para-aural

## Abstract

Mineralization of the para-aural soft-tissue region between the tympanic bulla, hyoid apparatus and nasopharynx in dogs has not been described in veterinary literature. The objective of the study was to determine the prevalence of para-aural mineralization on head computed tomography (CT), describe its morphological features, and assess associations with middle-ear disease, body weight, age, and breed. This retrospective case–control study of 1,833 canine head CTs (January 2012 – December 2024) identified para-aural mineralization in 15 dogs (0.8%) and affected cases were compared with 30 breed-matched controls. Affected dogs were predominantly male, median age 9.4 years and weight 27.3 kg, with lesions frequently bilateral, ventrolateral to the tympanic bulla, and having a median area 12.4 mm^2^ (median Hounsfield Units 424). All affected cases also showed external ear canal wall mineralization and 87% cf. 43% in the control cases had concurrent tympanic bulla disease (wsall thickening, lysis, and luminal soft tissue/fluid, or hyperostotic tympanic bone spicules) (*p* = 0.009), whereas age (*p* = 0.065) and weight (*p* = 0.709) did not differ significantly between the two groups. Dogs with para-aural mineral formations were more likely to have other soft tissue mineralization noted in the head CT. Cases with serial scans revealed coalescence of fragments, suggesting a dynamic calcification process. These CT findings indicate that para-aural mineralization is uncommon, but imaging findings could serve as a marker of occult chronic middle ear disease, rather than being associated with age or body weight.

## 1 Introduction

Middle ear disease encompasses a range of inflammatory and non-inflammatory conditions that have been well documented in dogs with a high incidence (50%) of otitis externa having secondary otitis media (1). Accurate diagnosis of middle ear disease can be challenging, particularly in patients that lack neurological signs or do not exhibit clinically significant otitis externa (2).

Advanced imaging modalities have become valuable in the diagnostic evaluation of the middle ear for conditions including otitis media, inflammatory polyps, cholesteatomas, and neoplasms (3–5). Radiography and ultrasonography have been employed for many years; however, they are limited by superimposition of surrounding bony and soft tissue structures (5, 6). In contrast, computed tomography (CT) has emerged as a preferred imaging modality due to its non-invasive diagnostic technique and superior ability to provide detailed cross-sectional views of the tympanic region without anatomical superimposition (3, 5, 7). Magnetic resonance imaging (MRI) also offers diagnostic value, particularly in assessing soft tissue structure pertaining to the external ear, inner ear and the brain (4, 8). Imaging findings in middle ear pathology, such as chronic otitis media, may include alterations in the shape or outline of the tympanic bulla, presence of abnormal tissue or fluid within the middle ear cavity, osteolysis, osteomyelitis, and involvement of the petrous portion of the temporal bone (3, 4, 9, 10). A known associated disease of chronic otitis media in dogs is the acquired form of middle ear cholesteatomas (11), with features identified on CT described (12, 13). Neoplasia originating in the middle or inner ear is rare and more commonly occurs as an extension from the external ear canal into the middle ear (14, 15).

Several studies report a variety of abnormalities of questionable clinical significance identified on CT involving mineralization of structures around the middle ear of dogs. These include hyperostotic tympanic bone spicules (HTBS) that appear on CT as “stalked bony globular structures” formed from osseous proliferations of small tympanic bone spicules (STBS), physiological bone growths in the septum bulla (16); and “well-defined, smooth, round, stone-like bodies imbedded in inflammatory tissue” that are mineral concretions of necrotic material within the tympanic bulla termed “otoliths” or “otolithiasis” (17). These structures have also been described in felines (cats (18), African lions (19), and other wild animal species (20)). Additionally, other reported non-expansile middle ear lesions of high-attenuation include osteoma (21) and exostoses (19). A more recent study investigated mineralization of the external ear canal wall of dogs on CT and found no correlation to chronic otitis externa, but a degenerative process was considered for the formation of these mineral lesions (22). Collectively, these lesions are small, non-destructive, and contained within the middle ear and external ear canal wall.

In CT studies at our hospital, we observed mineralization forming in the soft tissues between the tympanic bulla and hyoid bone which are a location that differs from the previously described mineralizing lesions of the tympanic bulla wall, the external ear canal wall, or the hyoid bone.

This retrospective study aims to better understand whether dogs who develop para-aural mineralization in this location are associated with concurrent ear disease or if these changes are a “normal variation” in some breeds. We hypothesized that changes would correlate with patient age and body weight, with older and larger dogs being more susceptible, but that they would be incidental findings with no clinical significance to a patient’s condition.

## 2 Materials and methods

### 2.1 Study permits

Ethics approval was provided by the Royal College of Veterinary Surgeons (RCVS) Ethics Review Panel (2022-098-Bain).

### 2.2 Study design

This is a retrospective, case–control, single center study on mineralization development of the para-aural region, between the tympanic bulla, hyoid bone and nasopharynx, in dogs at a United Kingdom (UK) referral hospital (Dick White Referrals, DWR). All diagnostic quality dog head CT studies acquired from January 2012 to December 2024 were collected from the DWR database. Patients were excluded if studies did not include the head from nose to at least C1. Each CT was evaluated to determine if mineralization was noted in the soft tissues of the para-aural region between the external ear canal and the nasopharynx. Patients were excluded if they had previously undergone ear canal surgery with mineralization only noted on the ipsilateral side, had severe bone destruction resulting in an inability to clearly identify the origin of mineralization, or had middle ear cholesteatoma. If multiple CT studies were obtained for a patient, only the first study where mineralization was first seen was included; serial studies of the patient were not evaluated as separate cases, however any descriptive observational changes to the mineralization over time were documented. A control group of breed matched dogs (twice the number for each breed) from within the cohort, which did not have mineralization, were selected for comparison of diagnosis and clinical signs. Patient’s age, breed, body weight, sex, presenting clinical signs and history, and final diagnosis were recorded.

All studies before May 2023 were acquired by PNMS MX (16 Slice) CT (Phillips Neusoft Medical Systems, Shenyang, China) and a MEDRAD KPM Stellant Workstation Power Injector (Bayer, Kaiser-Wilhelm-Allee 1, 51,373 Leverkusen, Germany). More recent studies were acquired by GE Revolution EVO (64 slice) CT (GE Healthcare, Cincinnati, Ohio, US) and OptiVantage dual injector system (LF Guerbet, 15 rue des Vanesses, Zone Paris Nord II, 93420 Villepinte, France). All patients underwent general anesthesia administered based on the attending anesthetist’s preference and were positioned in sternal recumbency for CT of the head. Images were acquired without contrast, and in some cases also following administration of iodinated intravenous contrast media, 2 mL/kg iohexol (Omnipaque 300 mg, GE Healthcare, Cincinnati, Ohio, US). Images were reconstructed using low frequency reconstruction algorithm (soft tissue window – window level 40, window width 300) and high frequency reconstruction algorithm (bone window – window level 600, window width 3,000) in the transverse plane to identify if mineralization and/or pathological changes pertaining to the structures of the head, particularly between the tympanic bulla, hyoid bones and nasopharynx.

### 2.3 Imaging analysis

Images were reviewed by two authors - one an ECVDI diagnostic imaging resident and the second an ECVDI board-certified radiologist. Both assessors were blinded when reviewing images. At the end of individual image review, both assessors met up to reach consensus. Only the high frequency reconstruction algorithm (bone window – window level 600 window width 3,000) was required for this study to identify the presence of para-aural mineralization. Multiplanar reconstruction (MPR) using a DICOM viewer (OsiriX MD, ver. 14.1.2; Pixmeo SARL, 266 Rue de Bernex – CH1233 Bernex – Switzerland) was used as necessary.

**Figure 1:**
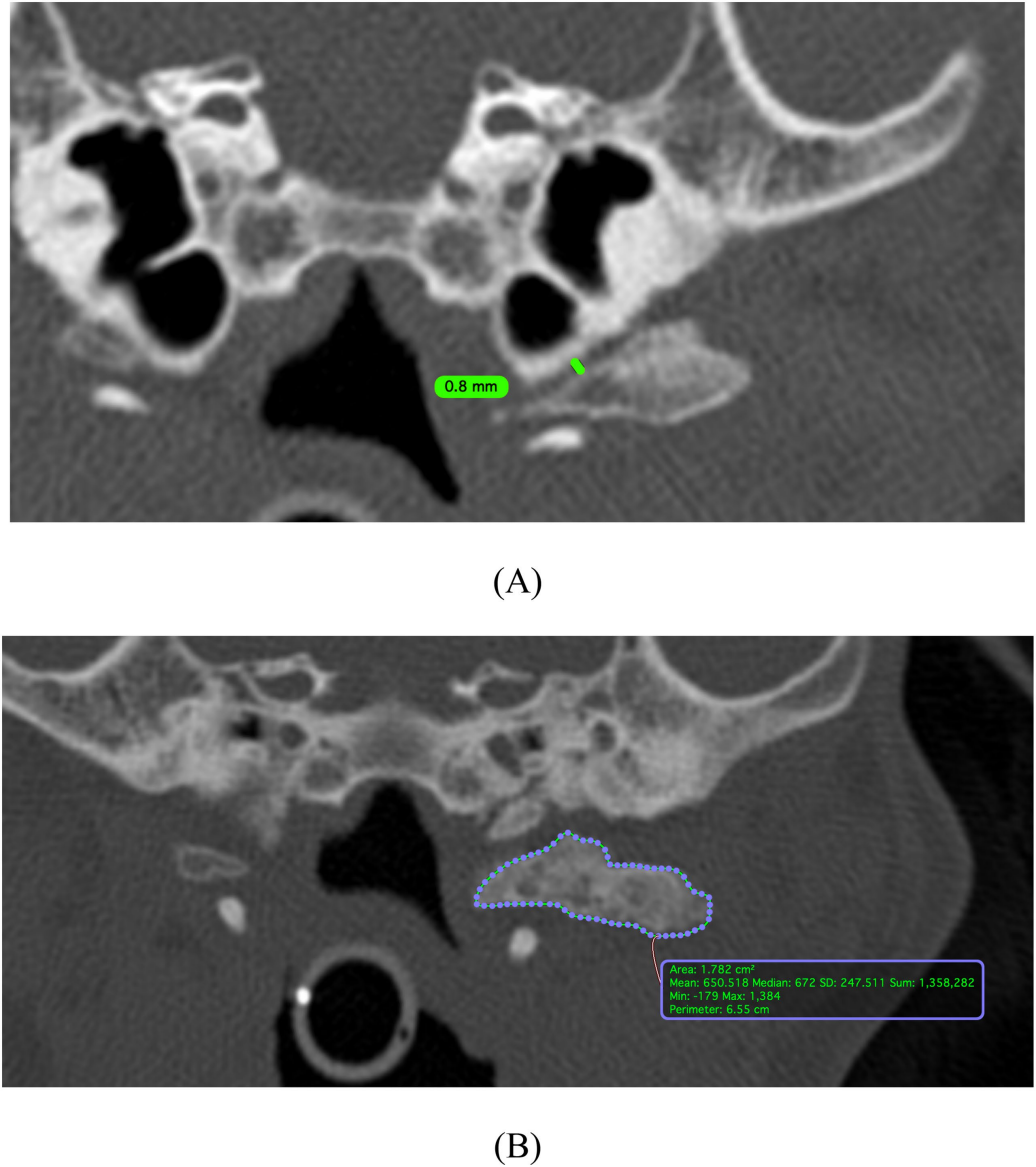
Image analysis of a case which met the inclusion criteria. High frequency reconstruction algorithm (bone window – window level 600 window width 3000) with para-aural mineralization having a shell-like appearance, located bilaterally (one focus on the left and two foci on the right), ventral and rostrally located in relation to the tympanic bulla, with the **(A)** closest distance being 0.8mm. **(B)** Placement of Hounsfield Units (HU) and area caliper for measurement of the largest para-aural mineralised fragment in transverse plane after multi-planar reconstruction (MPR) alignment along the longest axis of the sagittal and dorsal plane of the head.

Patients that met the inclusion criteria for the group with mineralization had the following documented about the Para-aural mineralization:

(1) Unilateral or bilateral.
(2) Location of mineral foci/focus in relation to the tympanic bulla: ventral, medial, ventromedial, lateral, ventrolateral, rostral, caudal. In some cases, multiple categories were allocated.
(3) Closest distance from the tympanic bulla (mm) (Figure 1).
(4) Number of mineral foci on the left and right side.
(5) The largest fragment was measured for Hounsfield Units (HU) and area in mm^2^ which was aligned along the longest axis in sagittal and dorsal plane in MPR with the area traced in the transverse plane (Figure 1).
(6) Described as having a shell-like appearance, linear or pin-point focus.

**Figure 2:**
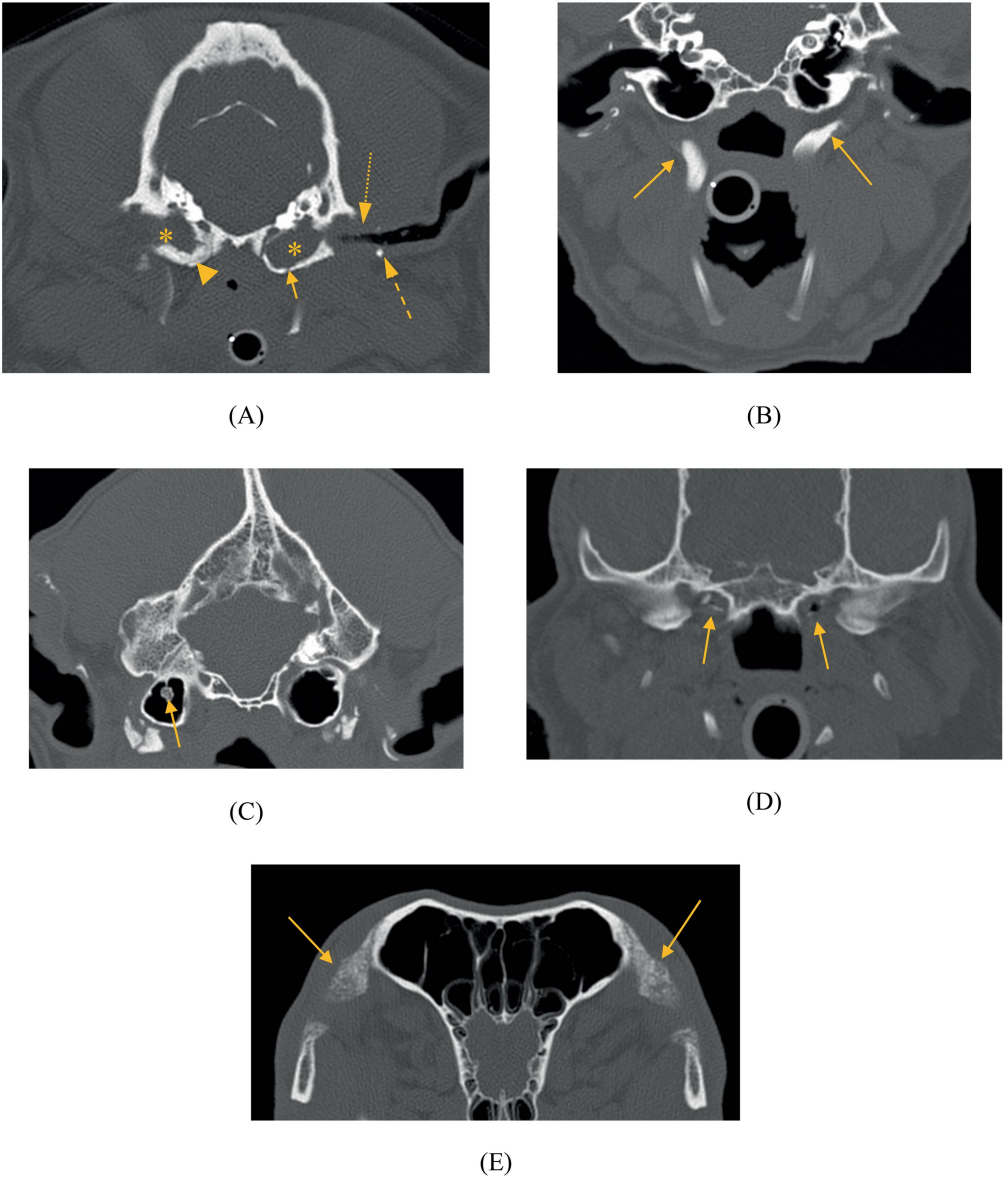
Abnormalities noted in the case group of the: **(A)** tympanic bulla – wall thickening (arrowhead), wall lysis (arrow), luminal soft tissue-fluid attenuating material (asterix); external ear canal wall mineralisation (dashed arrow) and luminal narrowing (dotted arrow), **(B)** hyoid – thickened stylohyoid bones (arrows), **(C)** hyperostotic tympanic bone spicule (arrow), **(D)** eustachian tube mineralisation (arrows), **(E)** orbital ligament mineralisation (arrows).

All patients and breed-matched control patients had the following documented (Figure 2):

(1) Tympanic bulla abnormalities:

a Unilateral or bilateral. For patients with unilateral para-aural mineralization, whether the tympanic bulla abnormality was contralateral or ipsilateral to it.
b Presence or absence of wall thickening, wall lysis, intraluminal soft tissue or fluid, HTBS or STBS.

(2) External ear canal assessed for the presence or absence of wall thickening, wall mineralization, and luminal narrowing.
(3) Hyoid abnormalities assessed as present (and described) or absent.
(4) Nasopharyngeal abnormalities assessed as present (and described) or absent.
(5) Other soft tissue or bone mineralization elsewhere in the head were described.

All measurements were taken in mm and rounded to 0.1, HU were recorded in whole numbers.

### 2.4 Statistical analysis

Data are summarized as median and range, or frequencies and percentages as appropriate. Comparisons between two groups were made using Fisher exact tests for categorical data, or Mann–Whitney tests adjusted for ties for continuous data. Significance was taken as *p* < 0.05. Analysis was undertaken in Minitab22 (Minitab, LLC., State College, Pennsylvania, USA).

## 3 Results

**Figure 3:**
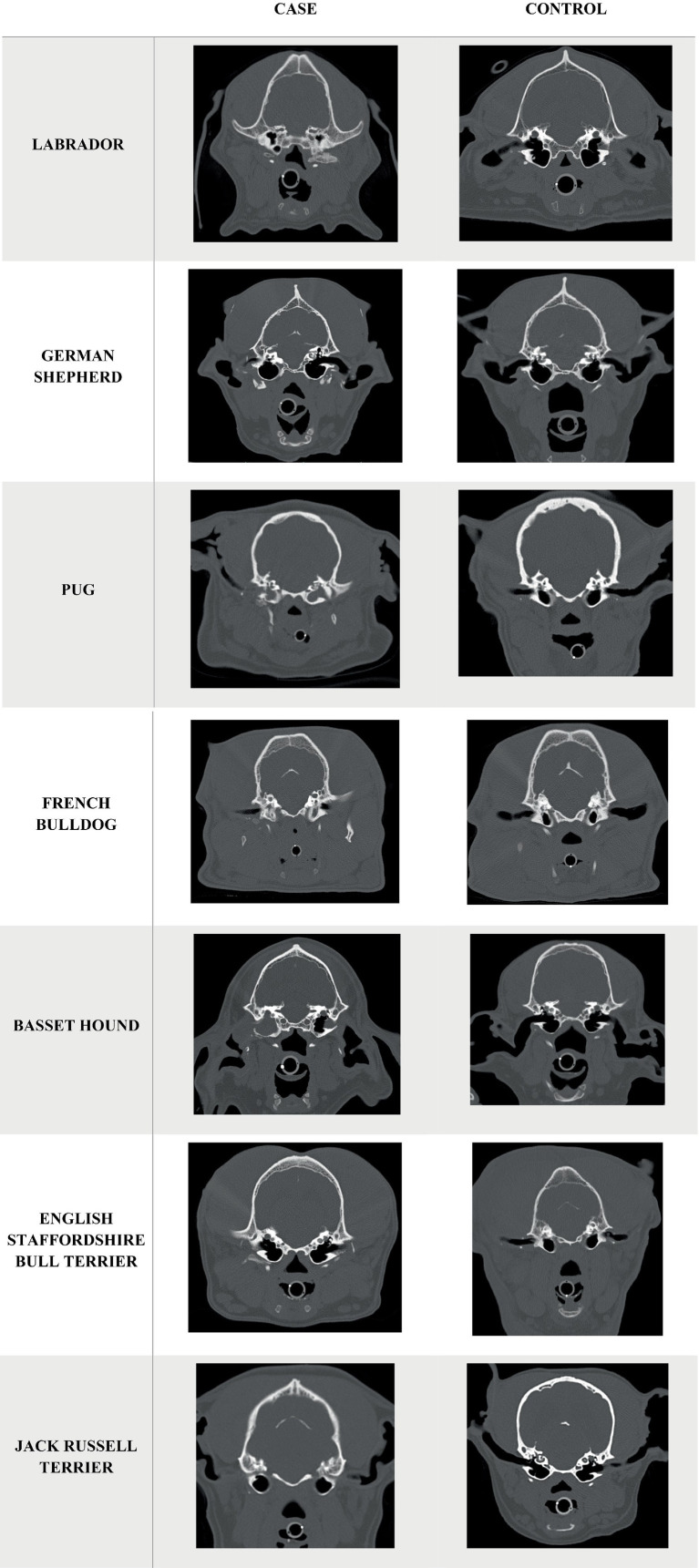
Transverse CT images of the head in high frequency reconstruction algorithm (bone window) showing para-aural mineralisation of the included cases. For each breed is a case and breedmatched control image.

From January 2012 to December 2024 there were 1833 dog head CT scans, 15 of which met the inclusion criteria. Breeds in the group with mineralization included five Labradors, three German Shepherds, two French Bulldogs, two Pugs, one Basset Hound, one English Staffordshire Bull Terrier, and one Jack Russell Terrier. Ten were male (four entire, six neutered), and five were female (one entire, four neutered). The median age was 9.4 years and ranged from 1.7 to 12.4 years. The median body weight was 27.3 kg with a range from 7.7 to 47.8 kg. The control group consisted of exactly double the number of each of the breeds in the case group. Fifteen were male (four entire, 11 neutered), and 15 were female (two entire, 13 neutered). Median age was 7.3 years (range 0.9 to 11.5 years). Median weight was 13.9 kg (range 6.6 to 46 kg). Age (*p* = 0.065) and weight (*p* = 0.709) did not differ significantly between case and control groups. The age, body weight and clinical diagnosis summary of each breed of the case group and control group are shown in Table 1, and images for each breed case and control showing the presence of para-aural mineralization in the case group are shown in(Figure 3).

**Table 1:** Patient signalment (breed, age, body weight) and relevant clinical history of the included 15 cases and 30 control dogs.

Breed	Number		Age	Body weight	Clinical diagnosis
Labrador	Case	5	Median 10.4 yr. Range 8.2–11.4	Median 33.3 kg Range 23.1–41.8	Low grade multilobular tumor, acanthomatous ameloblastoma, pleomorphic adenoma of the salivary gland, pterygoid soft tissue neoplasia with ramus osteophytosis, palatine osteolytic lesion suspected neoplasia
	Control	10	Median 8.7 yr. Range 1.1–11.4	Median 31.4 kg Range 26.4–38.2	Rhinitis, epithelial odontogenic tumor, cervical abscess/hematoma, oral acanthomatous ameloblastoma, fibrous gingival hyperplasia, masseter muscle soft tissue sarcoma, Anaplastic tumor of the submandibular lymph node, bilateral chronic otitis externa, nasal aspergillosis
German Shepherd	Case	3	Median 9.5 yr. Range 4.8–10.3	Median 42.4 kg Range 38.1–47.8	Unknown cause of unilateral epistaxis, unilateral mucopurulent nasal discharge, chronic otitis externa with unilateral tympanic bulla neoplasia
	Control	6	Median 5.5 yr. Range 4.1–7.4	Median 34.8 kg Range 25.9–46.0	Oral cavity foreign body, rhinitis, bilateral otitis externa, unknown cause of epistaxis, tonsillar SCC, penetrating stick injury
Pug	Case	2	Median 6.2 yr. Range 6.2–6.3	Median 10.2 kg Range 7.7–12.8	Bilateral otitis media (R TECA), BOAS with mild rhinitis
	Control	4	Median 9.4 yr. Range 3.1–11.5	Median 10.1 kg Range 8.1–15.5	Oral melanoma, unilateral rhinitis, left temporal muscle neoplasia
French Bulldog	Case	2	Median 5.6 yr. Range 1.7–9.4	Median 11.1 kg Range 10.1–12.1	Bilateral soft tissue nasal mass, bilateral otitis externa with amelanotic melanoma of the rostral lip
	Control	4	Median 5.9 yr. Range 3.8–9.0	Median 13.9 kg Range 10.5–14.5	medial pterygoid myositis, facial abscess, sialolith, sialadenitis, bilateral non-destructive rhinitis, BOAS
Basset Hound	Case	1	7.8	26	Aural inflammatory polyp of the middle ear
	Control	2	Median 5.0 yr. Range 0.9–9.0	Median 26 kg Range 23.8–28.2	Cervical foreign body abscess, bilateral chronic otitis externa
English Staffordshire Bull Terrier	Case	1	9.6	17.1	Inflammation/infection of nasal cavity
	Control	2	Median 8.5 yr. Range 7.3–9.7	Median 25.0 kg Range 23.6–26.5	Temporal abscess from foreign body, left rhinitis with nasal adenocarcinoma
Jack Russell Terrier	Case	1	12.4	11.4	Tracheal collapse
	Control	2	Median 6.4 yr. Range 1.2–11.5	Median 6.7 kg Range 6.6–6.8	Masseter and temporal myositis, jugular foramen syndrome/Vernet’s syndrome

### 3.1 Clinical history and diagnosis

The clinical history and diagnosis of the case group is as follows. All Labradors had tumors pertaining to structures of the head but not associated with the ear (low grade multilobular tumor, acanthomatous ameloblastoma, pleomorphic salivary gland adenoma, pterygoid soft tissue neoplasia, palatine osteolytic neoplasia). All German Shepherd cases had nasal disease and only one had tympanic bulla neoplasia with chronic otitis externa. The three included brachycephalic breeds (English Staffordshire Bull Terrier, French Bulldog, Pug) either had a history of inflammatory or non-inflammatory nasal disease, otitis media, otitis externa, or a nasal mass (round cell tumor). The Basset Hound and Jack Russell Terrier had an inflammatory middle ear polyp and tracheal collapse, respectively.

The clinical history and diagnosis of the control group is as follows. Four Labradors had neoplasia of the head but not associated with the ear (oral acanthomatous ameloblastoma, masseter muscle soft tissue sarcoma, and epithelial odontogenic tumor). The remainder of the Labradors had chronic neutrophilic lymphoplasmacytic rhinitis, nasal aspergillosis, fibrous gingival hyperplasia, cervical abscess/hematoma, or bilateral chronic otitis externa. The German Shepherds had foreign bodies in the oropharynx, non-specific rhinitis, bilateral otitis externa, unknown epistaxis and one tonsillar SCC. The Pugs had an oral melanoma, unilateral rhinitis, temporal muscle epithelial neoplasia and two cases had bilateral otitis externa. The French Bulldogs had medial pterygoid myositis, facial abscess, sialolith, sialadenitis, BOAS and one case having bilateral otitis externa. The Basset Hounds had foreign body abscess and bilateral chronic otitis externa. The English Staffordshire Bull Terriers had a nasal adenocarcinoma and foreign body abscess. The Jack Russell Terriers had myositis and Vernet’s Syndrome.

### 3.2 Para-aural mineralization

Para-aural mineralization in the case group was observed bilaterally (8/15, 53%) and unilaterally (7/15, 47%), with the majority of lesions located ventrolateral (12 dogs) to the tympanic bulla followed by rostral location (nine dogs), caudal (five dogs) and ventral (three dogs). Fragments had a median area of 12.4 mm^2^ (range 1–41 mm^2^) with HU median 424 (range 200–1,090). The patient with the largest fragment (41 mm^2^) was a 10.3 yr. male neutered German Shepherd with an unknown cause for mucopurulent nasal discharge. The closest distance from the tympanic bulla had a median of 0.8 mm (range 0.4–3 mm). The number of foci did not exceed five on either side. The left ear predominantly had one (six dogs) or no (five dogs) mineral focus, with only one dog having five foci. The right ear most frequently had two foci (seven dogs) followed by one (three dogs) or three (three dogs) focus/foci. Two dogs had serial CT scans with a decrease in the number of mineral foci but an increase in the area of the largest fragment, consistent with coalescing of fragments.

### 3.3 Tympanic bulla abnormalities

All but two case group dogs had unilateral or bilateral tympanic bulla abnormalities (13/15, 87%) with eight dogs (53%) having tympanic bulla wall thickening and two of these had wall lysis; seven (47%) had luminal soft tissue or fluid attenuating material, seven (47%) had HTBS. Six of the seven HTBS were in Labradors and German Shepherds. Two brachycephalic breeds (Pugs and French Bulldogs) in the study all had tympanic bulla wall thickening and intraluminal soft tissue or fluid.

Tympanic bulla abnormalities were observed in 13 of the 30 (43%) control group dogs; five of which had tympanic bulla wall thickening, three had intraluminal soft tissue/fluid material. None of the dogs had wall lysis. HTBS was present in 8 dogs (27%), all but 2 (one Jack Russell, one French Bulldog) were in dogs with a body weight >20 kg (Labradors, German Shepherds, English Staffordshire Bull Terrier).

From our population, there was a significant difference in the proportion of dogs in the case group with tympanic bulla disease compared to the control group (*p* = 0.009). There was significant association between the presence of para-aural mineralization and tympanic bulla thickening (*p* = 0.016), wall lysis (*p* = 0.032), and luminal soft tissue/fluid (*p* = 0.009) but no significant association with hyperostotic tympanic bone spicules (*p* = 0.200). Additionally, there was no significant association between unilateral or bilateral mineralization with tympanic bulla thickening (*p* = 1), tympanic wall lysis (*p* = 0.077), luminal soft tissue/fluid attenuating material (*p* = 0.619), or hyperostotic tympanic bone spicules (*p* = 0.315).

### 3.4 External ear canal abnormalities

All case group dogs had external ear canal wall mineralization, 13 (87%) of these had external ear canal thickening and 12 (80%) had luminal narrowing.

Of the control group, external ear canal wall mineralization was present in 22 dogs (73%). Thirteen dogs (43%) had both external ear canal wall thickening and luminal narrowing, 12 of which had wall mineralization; seven of these were of a brachycephalic breed.

There was significant association between the presence of para-aural mineralization and external ear canal thickening (*p* = 0.009), wall mineralization (*p* = 0.038), and luminal narrowing (*p* = 0.027) abnormalities.

### 3.5 Hyoid abnormalities

Only two case group dogs (13%) had hyoid abnormalities – one 8.2 yr. male entire Labrador diagnosed with a caudally located oral low-grade multi-lobular tumor had an irregular shape to the stylohyoid, irregular mineralization of the basihyoid, and irregular mineralization at the point where the hyoid contacts the larynx; one 10.3 yr. male neutered German Shepherd with an undetermined cause of nasal discharge had markedly thickened bilateral stylohyoid bones and no visible cortex and medulla, consistent with sclerosis.

No dogs in the control group had hyoid abnormalities.

There was no significant association between the presence of para-aural mineralization and hyoid abnormalities (*p* = 0.106).

### 3.6 Nasopharyngeal abnormalities

Eight case group dogs (53%) had nasopharyngeal abnormalities (three Labradors, three German Shepherds, one French Bulldog, one English Staffordshire Bull Terrier); all were bilateral eustachian tube mineralization.

Only three control group dogs (10%) had nasopharyngeal abnormalities, all of which were eustachian tube mineralization.

There was significant association between the presence of para-aural mineralization and nasopharyngeal abnormalities (*p* = 0.003).

### 3.7 Other mineralization elsewhere in the head

Lastly, nine case group dogs (60%) had mineralization elsewhere in the head (4 out of 5 Labradors, all German Shepherds, one Basset Hound and one Jack Russell Terrier). Seven of these had orbital ligament mineralization with one Labrador also had whiskers mineralization; two of the Labradors had epiglottis mineralization or osteochondrosarcoma of the left maxilla.

Only three control group dogs (10%) had mineralization elsewhere in the head; all with orbital ligament mineralization (two Labradors, one German Shepherd).

There was significant association between the presence of para-aural mineralization and other mineralization elsewhere in the head (*p* < 0.001).

## 4 Discussion

This retrospective case–control study documents the first CT description of para-aural mineralization within the soft tissue region between the tympanic bulla, hyoid bones and nasopharynx of dogs. Over a 13-year period, only 15 of 1833 head CT examinations met inclusion criteria. These cases revealed a predominance of males and a ventrolateral distribution to the tympanic bullae of para-aural mineral lesions with lesions presenting almost equally as unilateral or bilateral. There was a statistically significant association between para-aural mineralization and concurrent tympanic bulla disease; however it is not clear if this mineralization is an incidental degenerative process or if it may be an uncommon secondary manifestation of chronic inflammatory or (rarely) neoplastic process within the middle ear.

It was observed that mesocephalic dogs >20 kg in our study had HTBS which is consistent with previous studies reporting the prevalence of HTBS in high body weight patients (16). Despite the presence of HTBS, our study showed a lack of significant association between HTBS and para-aural mineralization suggesting these mineralizations are not related. Additionally, brachycephalic dogs in our study all had tympanic bulla wall thickening with intraluminal soft tissue or fluid attenuation, consistent with prior studies identifying Pugs and French Bulldogs having abnormal skull conformations and aeration of the auditory tube predisposing them to primary secretory otitis media (23–25). The presence of these bulla changes was significantly associated with the development of para-aural mineralization in our population. The statistical association and high frequency of external ear canal wall mineralization and frequent luminal narrowing in our study could suggest a chronic otitis-driven pathogenesis with potential dystrophic calcium deposition secondary to persistent inflammation. This differs from a more recent study which concluded from their population that external ear canal wall mineralization occurred independently of ear disease duration (*p* = 0.49) with mineralizations occurring in 80% (20/25) of disease-free, 78% (33/45) of chronic and 80% (8/10) of acute otitis externa – therefore suggesting a degenerative process should also be considered (22). Lastly, the observation of serial CTs showing coalescence of mineral fragments underscore the dynamic process rather than static calcification.

Limitations include the small sample size of the case group, retrospective design, and potential selection bias of the control group and also inherent bias in a single-institution CT database. Prospective studies with larger, multi-center cohorts and histopathologic correlation are warranted to clarify the etiologic mechanisms and to determine whether early detection of para-aural mineralization could serve as an imaging biomarker for underlying otic disease.

## 5 Conclusion

Contrary to our hypothesis that para-aural mineralization would correlate with increasing age and body weight and represent an incidental, clinically insignificant finding, no significant association with age or weight was identified, and the strong correlation with concurrent middle and external ear disease suggests these changes are pathologic rather than incidental. This study identifies para-aural mineralization as a rare but distinct CT finding in dogs, predominantly affecting middle-aged, larger breeds and presenting most often ventrolateral to the tympanic bulla. The strong association with concurrent tympanic bulla disease (*p* = 0.009) and the universal presence of external ear-canal mineralization suggest that these deposits arise secondary to chronic otic inflammation or neoplasia rather than representing age or weight related skeletal changes. Brachycephalic conformation predisposes to bulla wall thickening and intraluminal fluid, yet it does not increase the risk of para-aural mineralization, highlighting the likely underlying inflammatory process over skull morphology. The dynamic evolution of mineral fragments on serial imaging supports an active calcification process linked to ongoing disease. Although limited by sample size and retrospective design, the findings raise the possibility that para-aural mineralization could serve as an uncommon ancillary imaging marker for occult middle-ear pathology. Prospective, multi-center investigations with histopathologic validation are needed to clarify the pathogenic mechanisms, determine clinical relevance, and evaluate whether early detection influences therapeutic outcomes in dogs with chronic otitis media or aural neoplasia; or if a larger cohort will find these changes are incidental degenerative change.

## Data Availability

The original contributions presented in the study are included in the article/supplementary material, further inquiries can be directed to the corresponding author.
